# For many but not for all: the bikini incision direct anterior approach for total hip arthroplasty. A narrative review

**DOI:** 10.1186/s10195-024-00812-z

**Published:** 2024-12-18

**Authors:** Cesare Faldini, Francesco Traina, Federico Pilla, Claudio D’Agostino, Matteo Brunello, Manuele Morandi Guaitoli, Alberto Di Martino

**Affiliations:** 1 Department of Orthopedics and Traumatology, IRCCS Istituto Ortopedico Rizzoli, Via Giulio Cesare Pupilli 1, Bologna, Italy; 2 Department of Biomedical and Neuromotor Science—DIBINEM, University of Bologna, Bologna, Italy; 3 Orthopedics-Traumatology and Prosthetic Surgery and Hip and Knee Revision, IRCCS Istituto Ortopedico Rizzoli, Bologna, Italy

**Keywords:** Total hip arthroplasty, Minimally invasive, Bikini incision approach, Direct anterior approach, Hip, Lateral femoral cutaneous nerve

## Abstract

Total hip arthroplasty (THA) has significantly improved the lives of patients with degenerative hip disorders. The direct anterior approach (DAA) is favored for its minimally invasive nature, leading to less postoperative pain and a faster recovery. The bikini incision (BI) approach was developed to enhance aesthetic outcomes while maintaining the clinical and functional benefits of the DAA. Despite its advantages, the BI technique presents challenges, controversies persist regarding its efficacy and safety, and there is no consensus within the medical community about its overall benefits. Incisions aligned with Langer’s lines, like the BI, promote better healing and minimal scarring. Studies indicate that BI patients report higher satisfaction with scar appearance and texture compared to traditional DAA patients. However, the BI carries a higher risk of lateral femoral cutaneous nerve (LFCN) injury, although most symptoms resolve within 6 months. For obese patients, the BI is associated with fewer complications, such as infections and delayed healing, compared to the conventional DAA, making it a safe and effective option. BI patients also experience better aesthetic outcomes and functional recovery, with reduced pain and itching. The BI technique in THA represents a significant advancement, offering improved aesthetic and wound-healing outcomes. The shift from the traditional DAA to the BI aligns with patient preferences for scars that are less visible and conspicuous. Despite the steep learning curve and risks, careful patient selection and refined surgical techniques can enhance the BI’s benefits. Future research should focus on long-term outcomes and comparative studies to further establish the BI’s efficacy and safety. As patient demand for aesthetically favorable surgeries grows, the BI is likely to become a preferred approach in THA.

## Introduction

Total hip arthroplasty (THA) is a transformative procedure that has greatly reduced pain and improved the lives of those with hip joint disorders leading to degeneration [1, 2]. Among the various surgical techniques for THA, the direct anterior approach (DAA) has gained attention because it is a muscle-sparing method, working through a plane between the sartorius and tensor fascia latae muscles [3]. This minimally invasive approach is praised for reducing postoperative pain, promoting a quicker recovery, and lowering dislocation rates [4–7]. The DAA has a long history. It was first described by Carl Hueter in 1881 for treating hip infections and injuries, later refined by Smith-Petersen in 1917 for orthopedic use [8, 9], and subsequently pioneered for hip arthroplasty by Judet [10]. The modern DAA gained further popularity, especially in the US, after Matta highlighted its advantages in 2005 [3].

As THA techniques have evolved, there has been an increasing focus on the use of minimally invasive approaches to reduce complications and enhance cosmetic outcomes [11]. This has led to the development of the minimally invasive bikini incision (BI) approach, introduced by Leunig in 2013 as a refinement of the DAA. The BI approach offers both aesthetic and functional improvements over traditional methods [12]. It has shown excellent clinical and aesthetic outcomes [13–15], though concerns about its learning curve and complexity remain [16–18]. Additionally, some have raised concerns about higher postoperative complication rates associated with the BI technique [19–21].

Our aim is to provide a balanced analysis of the BI approach, exploring its technical aspects, benefits, challenges, and impact on patient outcomes. We discuss incision methods, aesthetic results, the learning curve, and patient selection criteria to offer a comprehensive view of this technique.

## Langer’s lines

**Fig. 1:**
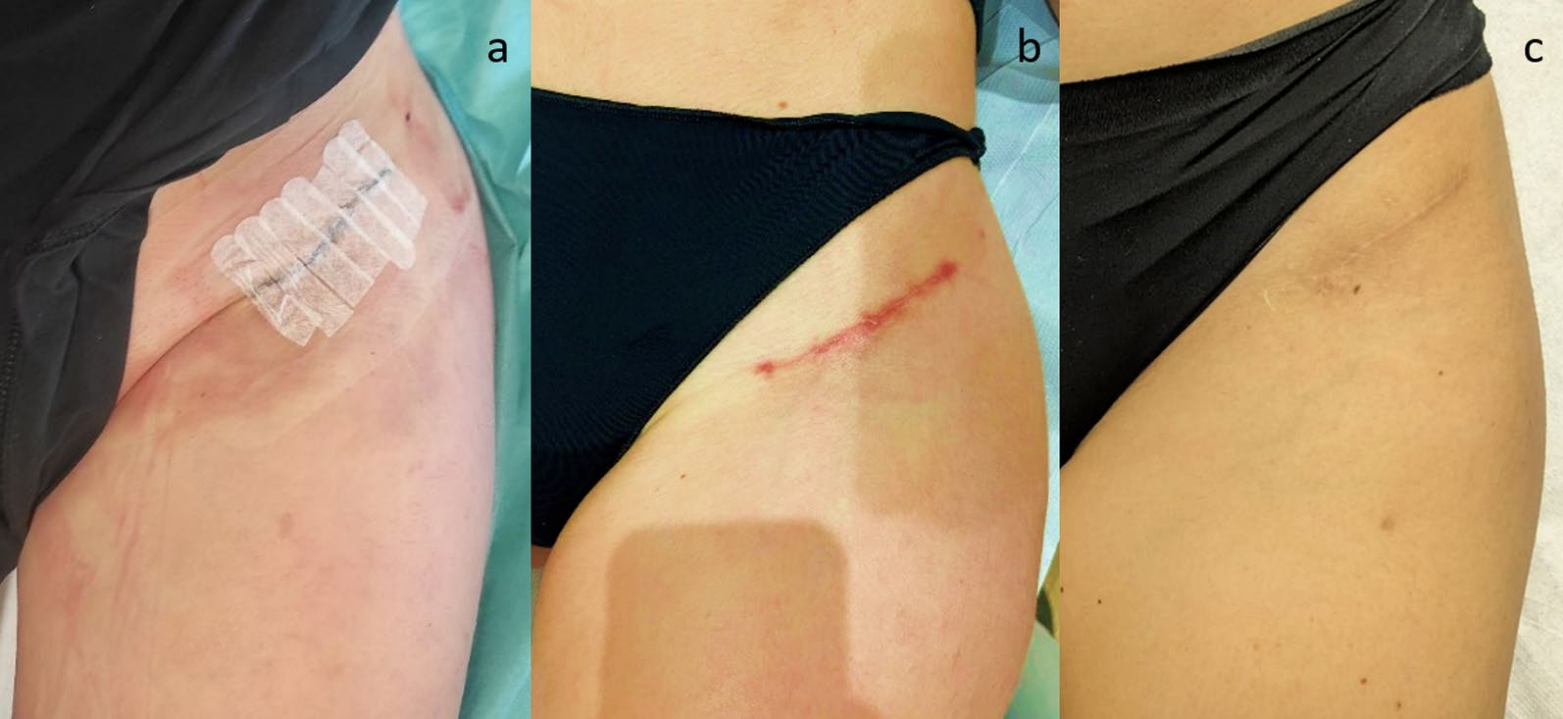
Images illustrating the different stages of surgical wound healing: at 7 days post-surgery (**a**), at 1 month post-surgery (**b**), and at 9 months post-surgery (**c**)

Since Karl Langer first identified the predominant directions of skin tension—known as Langer’s lines—in 1861, they have become an essential guide for surgical incisions. Initially seen as static and unrelated to scar healing [22], recent studies in plastic, craniomaxillofacial, and orthopedic surgery have renewed interest in their clinical importance. It is now understood that Langer’s lines are dynamic: they adjust their orientation during the healing process [23]. Incisions made along these lines, which align with the skin’s natural tension and collagen fibers, reduce tension at wound edges, minimize the risk of wound dehiscence, and promote better healing with minimal scarring [24, 25] (Fig. 1).

Research from vascular surgery supports the benefits of horizontal incisions in reducing wound complications and infections compared to vertical ones. A randomized controlled trial by Swinnen et al. [26] involving 88 patients (116 groins) found that vertical incisions had a rate of complications of 47.5%, compared to just 12.7% for horizontal incisions (*p* < 0.001). Additionally, fewer infections occurred with horizontal incisions (*n* = 3) compared to vertical ones (*n* = 10). This improved healing was linked to the alignment of horizontal incisions with Langer’s lines [26]. Building on the principle of respecting natural skin tension and Langer’s lines, Leunig introduced the BI as a modification of the DAA for hip replacement surgery [12]. This oblique incision, following the groin’s natural skin crease, was designed to enhance both subjective and objective aesthetic outcomes while preserving the precision of component placement and ensuring the overall safety of the procedure.

## Surgical technique

**Fig. 2:**
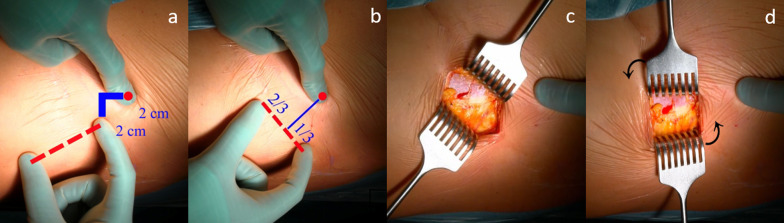
Starting from the ASIS, the surgeon moves 2 cm distally and laterally; this serves as the proximal reference point for the DAA incision (**a**). The imaginary line is then adjusted to run parallel to the iliac crest, positioning the starting point of the DAA incision two-thirds lateral and one-third medial to this line (**b**). The index finger is on the ASIS; the soft subcutaneous tissue is mobilized by the deep tissue, creating a “mobile window” (**c**). Moving the retractors vertically simultaneously, the mobile window is created in the direction of the muscle fibers, recreating the classical space for the standard DAA (**d**)

When performing the surgical technique for BI THA, patients are positioned supine [3, 27] on a standard radiolucent table or with the foot of the operated leg secured by a boot on a dedicated traction table managed by a non-scrubbed assistant, which allows control of the traction, rotation, and adduction or abduction. The patient’s lower limb is positioned with the leg at 20° of flexion, with neutral abduction and rotation. In the standard longitudinal DAA, the surgical incision is started 2 cm distally and 2 cm laterally from the anterior superior iliac spine and averages 7 cm [28]. In the BI, a 6- to 8-cm incision is made along Langer’s line at the groin fold, guided by an imaginary line perpendicular to the anterior superior iliac spine (ASIS) that crosses the inguinal fold [12]: the incision lies two-thirds lateral and one-third medial to this line, representing the projection of the standard DDA incision to the groin fold (Fig. 2).

After careful and smooth subcutis dissection, a plane is developed between the subcutis and the fascial plane; using two blunt retractors, the skin is rotated by 90° (replicating traditional DAA conditions), identifying the fascia over the tensor fascia latae and sartorius muscles. The rest of the surgery follows the standard DAA approach to the hip joint [27]. The fascia is incised over the tensor fascia latae muscle to minimize the risk of injuring the lateral femoral cutaneous nerve. The intermuscular and interneural space is then bluntly dissected: the tensor fascia latae is retracted laterally, while the sartorius and rectus femoris are retracted medially. Branches of the lateral circumflex artery are ligated or coagulated. The capsule is fully exposed and carefully opened, creating a thick flap that is sutured at the end of the surgery. The femoral neck osteotomy is performed in situ with an oscillating saw, and the head is removed using a corkscrew, with the leg in slight traction and external rotation.

Acetabular and femoral bones are prepared for implant positioning using dedicated instrumentation with curved and offset handles to ease bone preparation and avoid soft-tissue impingement. The iliofemoral and pubofemoral ligaments are incised to improve proximal femur exposure. Accurate posterior–medial capsular release at the proximal femur is performed before femoral broaching, with the patient’s leg in external rotation, extension, and adduction. After positioning the cup and femur implants, the reduction maneuver is performed, followed by suturing.

## Risks of lateral femorocutaneous nerve injury

The DAA is often criticized for its risk of neural damage, particularly to the lateral femoral cutaneous nerve (LFCN), which can result in temporary or permanent anesthesia, paresthesia, or dysesthesia in the anterolateral thigh [20, 29]. The most common neuropathic symptom following DAA total hip arthroplasty (THA) is numbness, reported in 15–37% of patients, though it is usually temporary [30–32]. The LFCN is a purely sensory nerve originating from the second and third lumbar nerves [33], and its variable anatomy can influence the likelihood of injury during surgery.

### Anatomical variability of the LFCN

**Fig. 3:**
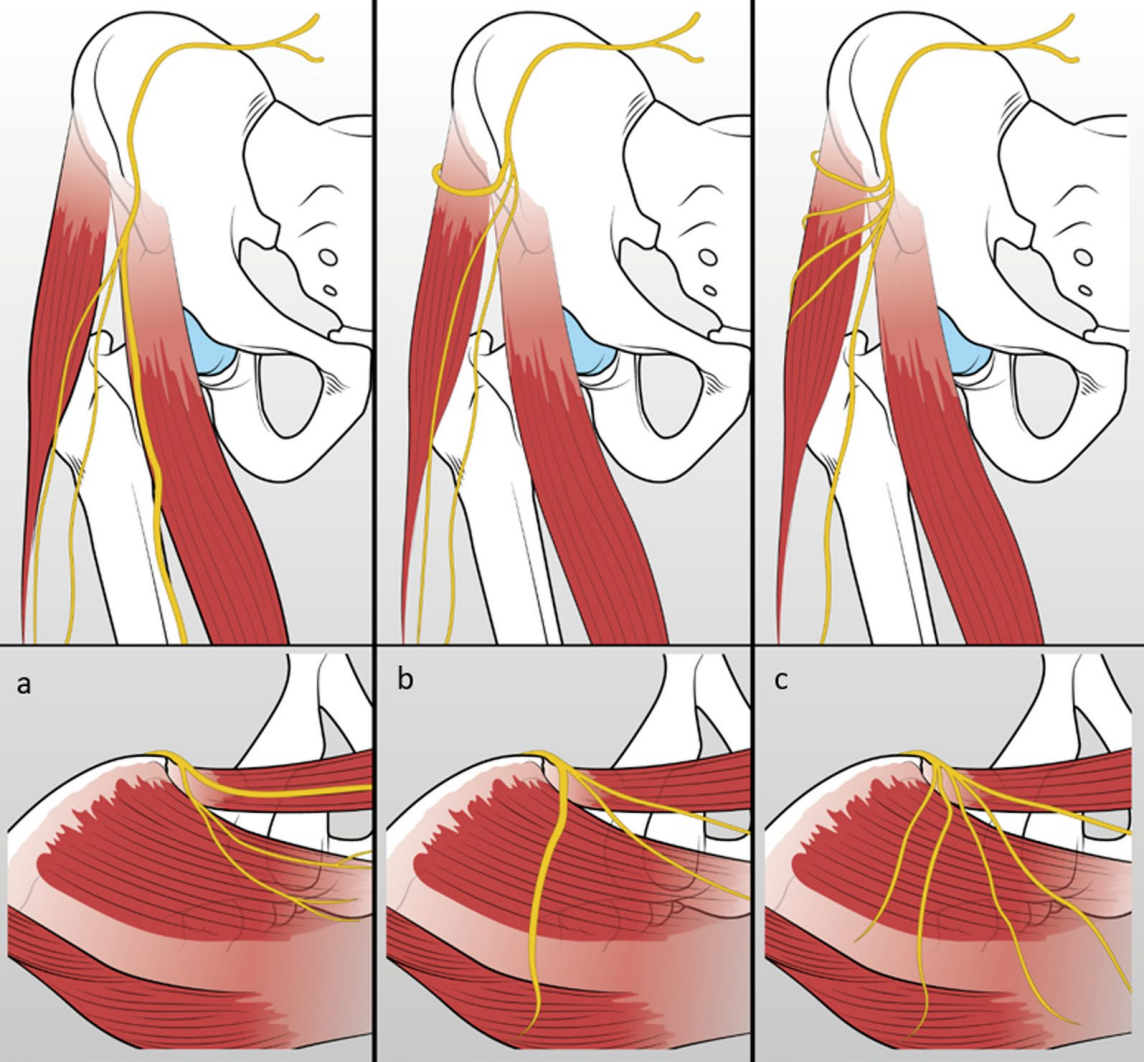
The different branching patterns of the lateral femoral cutaneous nerve within the thigh.** a** The sartorius type (36%, characterized by a dominant anterior branch running along the lateral side of the sartorius muscle.** b** The posterior type (32%), featuring a thick posterior branch running perpendicular to the belly of the tensor fasciae latae muscle.** c** The fan type (32%), characterized by multiple nerve branches spreading out with equal thickness

The LFCN’s course varies significantly, crossing the inguinal ligament approximately 1.4 cm medial to the ASIS and showing different branching patterns [34]. Rudin et al. [35] identified three main types of LFCN branching in cadaver studies. The sartorius type was observed in 36% of cases and features a dominant anterior branch that runs along the lateral border of the sartorius muscle. The posterior type occurs in 32% of cases and is characterized by a prominent posterior branch that crosses over the TFL muscle. The fan type, also present in 32% of cases, consists of multiple branches spreading across the anterolateral thigh and intersecting with both the TFL and sartorius muscles (Fig. 3).

### LFCN injury in BI vs. DAA THA: comparing study outcomes

The risk of LFCN injury has been examined in multiple studies comparing the BI with the standard longitudinal DAA. Sang et al. [19] conducted a randomized study involving 99 BI THA patients and 96 DAA patients, finding a higher incidence of LFCN injury in the BI group at 1.5 months post-surgery (36.4% vs. 21.9%, *p* < 0.05). Notably, while the initial rate of injury was higher for the BI, the majority of patients in both groups recovered over time, and by 6 months, there was no significant difference in persistent symptoms (7.1% for the BI vs. 4.2% for the DAA). This suggests that while the BI may pose a higher short-term risk, long-term outcomes are similar to the DAA. Sang et al. suggested that positioning the BI incision more laterally might reduce the risk of LFCN injury, particularly as more medial incisions or blunt dissection can increase vulnerability [19]. Supporting this, Thaler et al. [36] analyzed the relationship between various anterior approaches and LFCN patterns, noting that all types of skin incisions could affect the nerve, but the fan-like distribution of the LFCN branches in particular raised the risk for the BI. This finding reinforces the importance of careful incision placement to minimize nerve damage.

However, not all studies agree on the higher risk of LFCN injury with the BI. In a larger study of nearly 1,000 patients, Leunig et al. [13] found no long-term increase in hypoesthesia with the BI compared to the DAA. Over a 2- to 4-year follow-up, they noted similar rates of sensory disturbance between the two approaches, with approximately 15% of DAA patients and 8% of BI patients reporting hypoesthesia. Furthermore, less than 10% of these patients described the numbness as bothersome, suggesting that it did not significantly affect quality of life even when present. Adding to the mixed evidence, Butler et al. [37] conducted a systematic review focusing on LFCN injuries in the BI versus the DAA. Of the five relevant studies they analyzed, two reported no statistically significant difference between the approaches [12, 38], while two others found a higher incidence of LFCN injuries in BI patients, though without statistical significance [19, 39]. Leunig’s 2018 study, as previously mentioned, found significantly lower rates of hypoesthesia in BI patients (*p* < 0.001) [13]. These contrasting results indicate a lack of consensus in the literature regarding the frequency and severity of LFCN injuries in BI versus DAA THA.

### Risk factors and preventive strategies

While LFCN injury can affect patient recovery and quality of life, the symptoms often improve over time without functional limitations. Studies with an 8-year follow-up showed that the prevalence of numbness decreased to about 10% [30, 31]. The main causes of LFCN damage are often linked to prolonged compression by retractors rather than direct nerve injury. As such, surgical time and technique are crucial factors, with experienced surgeons being less likely to cause nerve compromise. Additionally, mechanical entrapment of the LFCN during fascia closure can lead to severe postoperative pain or long-term numbness [40]. To minimize the risk of LFCN injury, two key precautions are recommended: first, when the LFCN is visible, care should be taken to avoid it during suture placement; and second, if the nerve is not visible, sutures should be placed as laterally as possible to reduce the likelihood of entrapment.

### Treatment of LFCN injuries

For patients experiencing iatrogenic meralgia paresthetica, conservative treatment options like local anesthetic injections with or without steroids have shown good results. Ultrasound-guided injections allow for precise targeting of the nerve entrapment site, which can effectively alleviate symptoms [41]. If conservative treatment fails, surgical options like neurolysis or neurectomy may offer significant and lasting symptom relief [42].

## Periprosthetic fractures and the BI

Periprosthetic fractures are a well-recognized complication of THA performed via the DAA [43]. They present both functional challenges for patients and a significant socioeconomic burden due to increased hospitalization costs [44, 45]. A comparative study by Leunig et al. involving 964 patients found no significant difference in periprosthetic fracture rates between the BI and DAA groups (one fracture in the BI vs. two in the DAA group). These results are consistent with findings by Alva et al. [46], who reported similar frequencies of periprosthetic fractures in both approaches, with three trochanteric fractures (0.35%) and four femoral shaft fractures (0.47%) in BI patients.

Intraoperative femoral fractures may require immediate reduction, which can be challenging with a BI. Some authors recommend extending the transverse skin incision longitudinally (into a T-shape) to allow better access for cerclage wiring and subsequent fracture stabilization [46–48]. Alternatively, a second small incision over the fracture site can be made to minimize trauma, allowing the fracture to be anatomically reduced and stabilized.

## Obesity and bikini incision

**Fig. 4:**
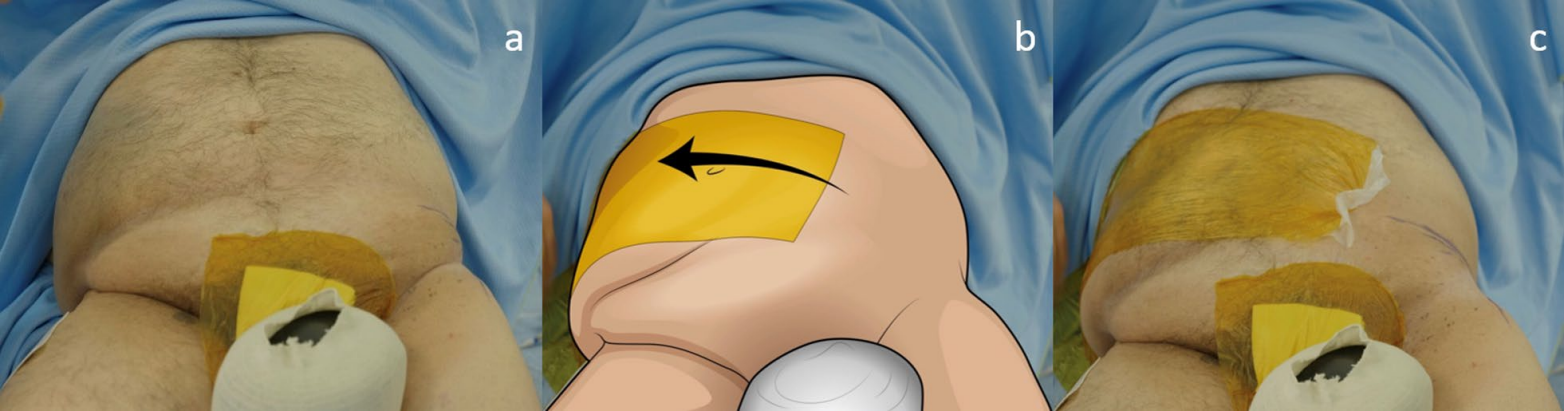
Images from left to right show the various stages of using a sterile drape to reduce the bulk of abdominal fat and enhance the visibility of the surgical field. **a** Abdominal fat covers the hypothetical surgical incision. **b** The drape is initially positioned on the ipsilateral side of the abdomen relative to the surgical site, and then it is drawn contralaterally to clear the operative field. **c** The surgical site appears free from adipose tissue thanks to the positioning of the sterile drape

Obesity is a growing concern in orthopedic surgery, as it is strongly associated with the increased incidence of end-stage osteoarthritis [49, 50]. This comorbidity poses challenges for THA, especially when using the BI. The pannus of fat present in obese patients can obstruct the operative field, making the BI more complex. As Corten et al. [47] suggest, it is crucial to assess the extent of skin covered by the pannus in the inguinal and anterior thigh regions, as these areas should be avoided during surgery to prevent complications like infections and wound dehiscence. Therefore, incisions should be placed away from the pannus without compromising access to the acetabulum and proximal femur. Intraoperative techniques, such as using a sterile drape to retract the pannus towards the contralateral side of the abdomen, can help maintain a clear surgical field (Fig. 4).

Despite these challenges, studies suggest that the outcomes of the BI in obese patients are comparable to those in non-obese patients. Manrique et al. [38], in a retrospective case–control study, found that obese patients (BMI > 30) who underwent the BI had significantly fewer wound healing complications (16.6% for the DAA vs. 0% for the BI, *p* < 0.05) and dysesthesia (6.3% vs. 0% for the BI) than those who had the conventional DAA. Additionally, obese patients in the BI group experienced shorter surgery durations and hospital stays, with no significant differences in estimated blood loss, intraoperative complications, or infection rates compared to non-obese patients.

These findings are consistent with those of Nizam et al. [51] and Verhaegen et al. [52]. Nizam et al. [51] concluded that the BI technique is both safe and effective for obese patients, with no significant differences in complication rates between obese and non-obese groups. Verhaegen et al. [52] similarly reported a reduction in wound complications among obese patients undergoing the BI, further supporting the clinical efficacy of this approach.

## Unsuitable patients

Certain patient profiles are less suitable for the BI approach due to specific anatomical or physiological challenges. Young, muscular patients, who tend to have hypertrophic muscles, present particular difficulties. The increased muscle bulk can make surgical exposure more challenging, leading to potential complications during the procedure [18, 53]. Additionally, patients with severe hip dysplasia or sequelae from conditions like Perthes disease pose unique anatomical challenges. These conditions often involve an altered acetabular and femoral anatomy, such as abnormal bony coverage, valgus or varus deformities, and changes in femoral anteversion. These anatomical differences may necessitate a wider surgical exposure, requiring the surgeon to extend the incision longitudinally, which is not compatible with the BI approach, particularly if additional procedures are needed for the acetabulum or femur [54, 55]. Patients requiring revision arthroplasty are also generally unsuitable for the BI. Revision surgeries often involve substantial scar tissue, altered bone anatomy from previous surgeries, and the need for extensive dissection to address implant failures or bone loss. In these cases, a more extended surgical approach is required, which the BI does not provide. While it is possible to convert the BI into an ilioinguinal approach for better exposure, this modification still limits the surgeon’s access to the proximal femur, complicating revision procedures. Similarly, patients with previous trauma treated with internal fixation devices like cephalomedullary nails or plate and screw systems are not good candidates for BI THA. The presence of pre-existing longitudinal scars often necessitates a more extensive incision, making the BI unsuitable for these cases.

## Learning curve

The concept of a learning curve in surgery refers to the stages a surgeon experiences as they acquire proficiency in a new technique. These stages typically include: (1) a rapid improvement phase during early training, (2) a period of slower progress as experience increases, (3) a plateau where additional experience has little effect on outcomes, and (4) a late decline due to factors like age-related reductions in manual dexterity or eyesight [56, 57].

For the DAA in THA, numerous studies confirm its safety and effectiveness, largely due to its muscle-sparing nature, which leads to less postoperative pain and quicker rehabilitation [58, 59]. However, the DAA is technically demanding, especially for surgeons with limited experience, and the learning curve is steeper than for other approaches. Nairn et al. [60] conducted a systematic review revealing that the steepest improvement occurs in the first 30 surgeries, and a plateau is reached at around 100 procedures. Operative time also decreased significantly during these first 30 cases, with times approaching those of more traditional approaches after about 50 surgeries [60]. Similarly, Masonis et al. [61] confirmed a reduction in surgical time after 100 cases, an important factor in reducing infection rates [62–64]. Complication rates are also linked to the surgeon’s experience. In Nairn et al.’s review, early DAA patients experienced an average complication rate of 20.8%, which dropped to 7.6% as surgeons became more proficient [60]. Revision rates and implant survival also improved with experience. Surgeons who had performed over 100 DAA cases achieved outcomes comparable to those seen with other approaches, with a revision rate of 1.1% [60]. De Steiger et al. [65], found that surgeons who had performed between 51 and 100 DAAs had a cumulative revision rate of 3% at 4 years, while those with more than 100 procedures had a rate of 2% (*p* = 0.33). Peters et al. [66], in a study of 15,875 THAs, observed that patients treated by surgeons who had performed no more than 50 DAAs had a 64% higher risk of revision. However, the complication rate began to stabilize and decrease after 100 surgeries, suggesting that the learning curve for DAA THA is approximately 100 cases [66, 67].

Although no specific studies have addressed the learning curve for the BI, it is reasonable to assume that it mirrors the DAA learning curve. Surgeons must first master the DAA, and once they achieve proficiency after about 100 cases, they can transition to the BI to ensure optimal outcomes and maintain patient safety.

## Aesthetic outcomes

Minimally invasive approaches and improved scar aesthetics are increasingly emphasized in orthopedic surgery. The BI offers favorable aesthetic outcomes compared to traditional longitudinal incisions, as horizontal incisions tend to promote better wound healing and reduce scarring [38, 39].

This has been demonstrated in multiple studies. For instance, Leunig et al. [12] observed superior cosmetic outcomes with the BI after 6 months, and their 2018 study further confirmed these findings, with patients reporting lower average scores on the University of North Carolina’s “4P” scar scale for itching, pain, paresthesia, and scar texture (0.2 BI vs. 0.4 DAA; *p* = 0.01) [13]. Wang et al. corroborated these results, finding that BI patients had significantly better SCAR scores at 6 months postoperatively compared to DAA patients (7.4 ± 1.8 vs 9.3 ± 2.0, respectively; *p* < 0.001) [39]. Di Martino et al. also reported superior scar quality in BI patients who took the POSAS questionnaire, as they gave significantly lower scores than those observed in both DAA and posterolateral approach (PL) groups [14.1 ± 3.9 (*p* = 0.021) and 14.5 ± 3 (*p* = 0.0032), respectively] [68]. Additionally, Jin et al. [14] found that BI patients had shorter incision lengths (9.7 ± 1.6 mm vs. 10.8 ± 2.0 mm; *p* < 0.01) and higher postoperative satisfaction compared to those who underwent the PL for bilateral THA.

## Conclusions

The BI represents a significant evolution in surgical technique for primary total hip arthroplasty (THA), offering both aesthetic and clinical benefits. It not only enhances the appearance of surgical scars but also promotes better wound healing by aligning the incision with the body’s natural contours and Langer’s lines. This shift from traditional longitudinal DAA incisions to the BI reflects a broader surgical philosophy aimed at maximizing both functional recovery and patient satisfaction, particularly in terms of cosmetic outcomes.

Studies consistently report that the BI yields favorable results regarding scar appearance, with patients experiencing higher satisfaction and fewer scar-related complications compared to traditional DAA incisions. The approach minimizes tissue trauma, respecting the skin’s elastic properties, and it supports a more physiological healing process. As societal preferences evolve, particularly regarding aesthetics, it is likely that more young, fit individuals as well as older patients will increasingly request the BI for THA, following trends observed in other surgical specialties where scar appearance influences patient choice [69, 70].

However, important challenges remain. The learning curve for the BI is still undetermined, though research suggests that 100 DAA procedures are necessary for surgeons to achieve proficiency and reduce complication rates [60, 65, 66]. As the BI requires similar expertise, ongoing training and education are essential to ensure successful outcomes. Surgeons must also be mindful of specific risks, such as LFCN injury and diaphyseal fractures, which demand careful management and surgical precision.

Future research should focus on overcoming these challenges, paying particular attention to long-term functional outcomes and complication rates associated with the BI in THA. Comparative studies with traditional approaches could provide valuable insights into its efficacy and safety.

In conclusion, the BI is a promising advancement in anterior THA, offering a cosmetically superior option without compromising early functional recovery. Although further research and clinical experience are needed, the demand for the BI in THA is expected to grow, and surgeons proficient in the DAA should prepare for more complex cases. Clear communication with patients regarding expectations and potential risks, such as the need for additional incisions in the event of complications, is essential. Ultimately, careful patient selection, taking into account individual risks and benefits, will enhance the potential of the BI to become a preferred approach in THA.

## Data Availability

Not applicable.

## Abbreviations

THA: Total hip arthroplasty
DAA: Direct anterior approach
BI: Bikini incision
LFCN: Lateral femoral cutaneous nerve
ASIS: Anterior superior iliac spine
PL: Posterolateral approach
